# Concurrent temporal channels for auditory processing: Oscillatory neural entrainment reveals segregation of function at different scales

**DOI:** 10.1371/journal.pbio.2000812

**Published:** 2017-11-02

**Authors:** Xiangbin Teng, Xing Tian, Jess Rowland, David Poeppel

**Affiliations:** 1 Max-Planck-Institute, Frankfurt, Germany; 2 New York University Shanghai, Shanghai, China; 3 NYU-ECNU Institute of Brain and Cognitive Science, NYU Shanghai, Shanghai, China; 4 School of Visual Arts, New York, New York, United States of America; 5 Department of Psychology, New York University, New York, New York, United States of America; University of Birmingham, United Kingdom of Great Britain and Northern Ireland

## Abstract

Natural sounds convey perceptually relevant information over multiple timescales, and the necessary extraction of multi-timescale information requires the auditory system to work over distinct ranges. The simplest hypothesis suggests that temporal modulations are encoded in an equivalent manner within a reasonable intermediate range. We show that the human auditory system selectively and preferentially tracks acoustic dynamics concurrently at 2 timescales corresponding to the neurophysiological theta band (4–7 Hz) and gamma band ranges (31–45 Hz) but, contrary to expectation, not at the timescale corresponding to alpha (8–12 Hz), which has also been found to be related to auditory perception. Listeners heard synthetic acoustic stimuli with temporally modulated structures at 3 timescales (approximately 190-, approximately 100-, and approximately 30-ms modulation periods) and identified the stimuli while undergoing magnetoencephalography recording. There was strong intertrial phase coherence in the theta band for stimuli of all modulation rates and in the gamma band for stimuli with corresponding modulation rates. The alpha band did not respond in a similar manner. Classification analyses also revealed that oscillatory phase reliably tracked temporal dynamics but not equivalently across rates. Finally, mutual information analyses quantifying the relation between phase and cochlear-scaled correlations also showed preferential processing in 2 distinct regimes, with the alpha range again yielding different patterns. The results support the hypothesis that the human auditory system employs (at least) a 2-timescale processing mode, in which lower and higher perceptual sampling scales are segregated by an intermediate temporal regime in the alpha band that likely reflects different underlying computations.

## Author summary

Correctly perceiving behaviorally significant sounds—speech, music, and the acoustic environment—requires integrating acoustic information over time to extract relevant regularities. A fundamental question about this process is: How does the auditory brain integrate information of continuously varying sounds, typical of many natural auditory signals? How does the brain “sample” the input? To investigate this question, we measured how cortical activity is entrained by sound using the noninvasive technique magnetoencephalography. We presented sounds with temporal structure at different timescales and examined how the brain encodes such signals. We found, unexpectedly, that the human auditory system does not treat all rates equally but predominantly uses 2 nonoverlapping timescales, the slower (theta) and faster (gamma) bands, to track acoustic dynamics, while the timescale corresponding to an intermediate (alpha) timescale is likely reserved for other cortical operations, perhaps relating to attention and functional inhibition. The data support the hypothesis that the human auditory system employs (at least) a 2-timescale processing mode and that the perception of natural sounds, which feels seamless and continuous, is underpinned by segregated and discontinuous neural processing.

## Introduction

Speech, music, and many natural sounds have a rich temporal structure over multiple timescales [1–5]; such sounds contain perceptually critical information that is encoded over short periods (e.g., the identity and exact sequence of phonemes in a spoken word) and, concurrently, information encoded over longer periods (e.g., the intonation change over a word that signals intent or affect). Successful perceptual analysis of these signals requires the auditory system to extract acoustic information at multiple scales. This presents a specific problem: how does the auditory system process different, co-occurring rates of information across multiple timescales? And, by extension, how can the requirements on temporal and spectral resolution simultaneously be met? To derive the appropriate perceptual representations, the auditory system must extract rapidly varying information on a scale of milliseconds (approximately 10–50 ms), operating with high temporal resolution, and concurrently analyze more slowly varying signal attributes on a scale of hundreds of milliseconds (about 150–300 ms), enabling sufficient spectral resolution [6]. A strictly hierarchical model, starting, say, with short/small integration windows at more peripheral processing regions, which are then concatenated to build longer windows, cannot by itself meet the perceptual demands [7–9].

Behavioral research suggests that the human auditory system may optimize processing by operating within separate temporal ranges instead of in a unitary way across a continuum of temporal variation [10–15]. Timescales on the order of tens of milliseconds are argued to be optimized for rapid temporal integration, such as in modulation detection [16], gap detection [17], and nonsimultaneous masking [18]. On the other hand, previous models of temporal integration typically assuming leaky integration demonstrate timescales above 150 ms, such as loudness summation [19,20], signal detection in noise [21,22], and temporal integration at threshold [14,23–26].

Here, we investigate whether these very clear behavioral results arguing for distinct timescales could be illuminated, especially in terms of their neural implementation, by considering cortical oscillations. Cortical oscillations reflect rhythmic activity of neural populations at different scales [27,28], and the frequencies of cortical oscillations are thought to reveal the corresponding temporal characteristics of sensory processing [29–32]. Therefore, by hypothesis, neural activity in the human auditory system may show a multiscale oscillatory pattern, which could reflect temporal scales of auditory processing. A short processing timescale, which guarantees high temporal resolution and may reflect the extraction or decoding of fine-grained acoustic information, could be reflected in the gamma band of oscillations that tracks fast acoustic dynamics, 30–50 Hz. A longer processing timescale, which integrates acoustic information on a timescale of 150–300 ms, could be reflected in the theta band oscillation, 4–7 Hz.

Of course, sounds contain information in ranges lying between these well-separated temporal regimes. Is there really such a compelling segregation of function in the time domain? Apart from the 2 timescales mentioned above, cortical oscillations within the alpha band (8–12 Hz) have been found in many studies to critically correlate with auditory attention [33–35], auditory working memory [36,37], and listening effort [34,38] and, furthermore, to predict speech intelligibility under challenging environments [39], which demonstrates a significant role for the alpha band in auditory processing. It is reasonable to hypothesize that the alpha band is fundamental to auditory perception, as demonstrated in a range of studies, but the timing (i.e., its intermediate position between lower theta and higher gamma activity) also invites the conjecture that alpha range activity reflects aspects of auditory processing on a timescale of approximately 100 ms. Here, we investigate directly the most straightforward hypothesis: does alpha band activity reflect the processing of acoustic information in a manner parallel to lower and higher neural frequencies?

Studies using amplitude-modulated sounds or click trains have shown a low-pass modulation transfer function (MTF) with a rebound above 30 Hz [40–45]. Although these findings demonstrate dominant auditory responses in the low frequency range (delta-theta and alpha) and the gamma band, the cortical oscillations entrained by stimuli of corresponding frequencies of the regular modulation are not sufficient to demonstrate the importance of theta, alpha, and gamma bands in auditory processing, because the entrainment could simply reflect a modulation-frequency following response which may have little to do with the auditory system actively processing acoustic information in the theta and gamma bands.

In studies using speech stimuli with irregular and complex modulations, it has been found that theta band activity entrains to the envelope of speech (and other signals), and that phase locking in the theta band is enhanced by increased speech intelligibility [46–54]. Different evidence suggests that the gamma band also plays an important role in phonemic and syllabic processing and comprehension of speech [55–60]. The results from speech processing suggest that theta band oscillations, instead of being simply entrained by modulations of the corresponding frequency, may actively “chunk” complex acoustic signals at a timescale corresponding to periods of the theta band [61,62], while the gamma band is involved in processing detailed information, because the comprehension of speech and syllable processing requires access to acoustic information at the phonemic scale.

Here, we aimed to elucidate what kind of mechanism might form the basis of such multiscale hearing by using irregular temporal modulations of auditory signals to ask whether neural oscillations are entrained equally to auditory stimuli of different irregular modulation rates or, rather, only specific, restricted bands; moreover, we asked whether different stimulus modulation rates can be decoded from specific neural frequency bands. We focus on cortical oscillations in the theta and gamma bands as well as the alpha band, in which strong effects related to auditory processing have been reported [63,64].

**Fig 1:**
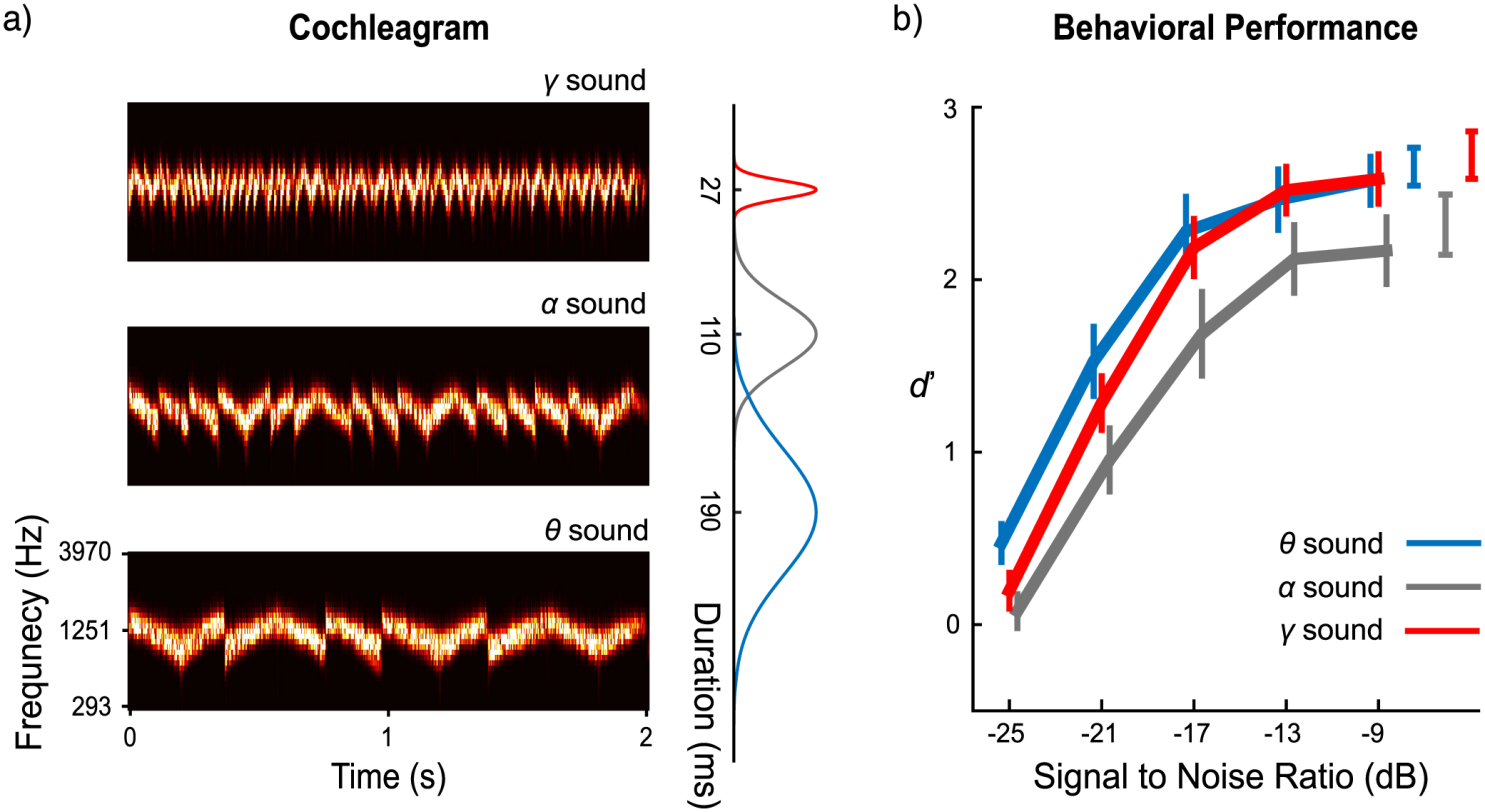
Stimuli and behavioral results. (a) Cochleograms of 3 stimulus types. A Gammatone filterbank of 64 banks was used to decompose the stimuli. The prior distributions of segment duration for the 3 stimuli are shown on the right. (b) Behavioral performance. The blue, gray, and red lines show d-prime values for *θ*, *α*, and *γ* sounds across different signal-to-noise ratios (SNRs), respectively. The 3 separately plotted results at the top-right corner are d-prime values in clean stimulus conditions. Error bars: ±1 standard error of the mean. Data are deposited in the Dryad repository: http://dx.doi.org/10.5061/dryad.f357r [121].

Building on experiments by Boemio et al. (2005) [65] and Luo and Poeppel (2012) [66], we generated acoustic stimuli with modulation rates that were centered at the typical periods of the neural theta (4–7 Hz), alpha-beta (8–15 Hz), and gamma (31–45 Hz) frequency bands (See Fig 1a for illustration of the stimuli). We measured using magnetoencephalography (MEG) the robustness of neural responses evoked by the stimuli and examined the correlations between the neural responses and the acoustic structure of the stimuli. We manipulated the signal-to-noise ratio (SNR) of the stimuli and evaluated the behavioral relevance of oscillations at the different rates. Next, classification analyses of the MEG data were performed to test which components of the neutrally elicited MEG signal were informative about the auditory signals. We show, contrary to expectation, that different temporal rates of the neurophysiological signal are differentially related to the behavioral, classification, and neural results. We further support the findings of processing at distinct timescales by performing mutual information analyses. Our combined neurophysiological and psychophysical results point to oscillatory neural mechanisms that underlie the segregated and discontinuous multiscale auditory processing of signals which, subjectively, feel seamless and continuous. In particular, we argue that the neural computations reflected in theta and gamma band activity differ in a principled way from those reflected in the alpha band and that alpha “splits” auditory processing into paired low and high processing scales.

## Results

### Differential sensitivity to varying modulation rates revealed by behavioral results

We first examine how well listeners can recognize stimuli with varying frequency modulation rates, including especially rates that correspond to theta, alpha, and gamma band periods. The behavioral sensitivity to different modulation rates provides a first indication whether specific rates are preferred by the human auditory system.

We refer to the stimulus type with a modulation rate in theta band range (4–7 Hz) as a theta (θ) sound, the stimulus type with modulation rate in the alpha band range (8–12 Hz) as an alpha (*α*) sound, and the stimulus type with modulation rate in the gamma band range (30–45 Hz) as a gamma (γ) sound. The cochleograms of 3 sounds are shown in Fig 1a. In the behavioral test, participants were asked to categorize the *θ*, *α*, and *γ* sounds by button press. Sounds were presented either as clean stimuli or presented in noise at various SNRs. The results (Fig 1b) demonstrate that participants identified all clean stimuli well (all d′ > 2) and that performance deteriorated in all conditions as the SNR level decreased. Particularly noteworthy is the reduced performance in correctly identifying the *α* sounds across all levels compared to both *γ* and *θ* sounds.

The behavioral performance in identifying the 3 clean stimuli was analyzed using a 1-way repeated measures ANOVA (rmANOVA) with Stimulus-type as the main factor. There is a main effect of Stimulus-type (*F*(2,28) = 9.78, *p* = 0.001, η_p_^2^ = 0.411). Planned comparisons using paired *t* tests reveal that the identification of *α* sounds was significantly worse than the identification of the other 2 stimuli (comparison to *θ* sound, *t*(14) = −3.16, *p* = 0.021, *d* = −0.82; comparison to *γ* sound, *t*(14) = −4.95, *p* = 0.001, *d* = −1.28; Bonferroni corrected). There was no difference in identification performance between the *θ* and *γ* sounds (*t*(14) = 0.65, *p* > 0.05, *d* = 0.17).

For the masked stimuli, a Stimulus-type × SNR 2-way rmANOVA reveals main effects of Stimulus-type (*F*(2,28) = 17.60, *p* < 0.001, η_p_^2^ = 0.557) and SNR (*F*(4,56) = 144.86, *p* < 0.001, η_p_^2^ = 0.912) as well as a Stimulus-type × SNR interaction (*F*(8,112) = 2.47, *p* = 0.017, η_p_^2^ = 0.150). A quadratic trend analysis indicates that identification of stimuli decreased as SNR decreases (*F*(1,14) = 510.35, *p* < 0.001, η_p_^2^ = 0.973).

### Phase coherence suggests differentiation between temporal regimes

We computed the intertrial phase coherence (ITC) across all frequencies from 2 Hz to 50 Hz and in a time range from 300 ms to 1,800 ms after the onset of the stimuli to measure how oscillatory cortical activity in each frequency band responds to different modulation rates. ITC measures the robustness of neural responses to stimuli across trials. If cortical oscillations at a certain frequency band reliably respond to a specific stimulus (and therefore, by hypothesis, probably encode information about this stimulus), we would obtain high ITC values at this frequency band. We created a distribution of ITC values by randomizing the onset time of each trial to normalize ITC in each subject across all frequencies and we converted the original ITC to *z*-scores of ITC (zITC). (See Materials and methods for details.)

**Fig 2:**
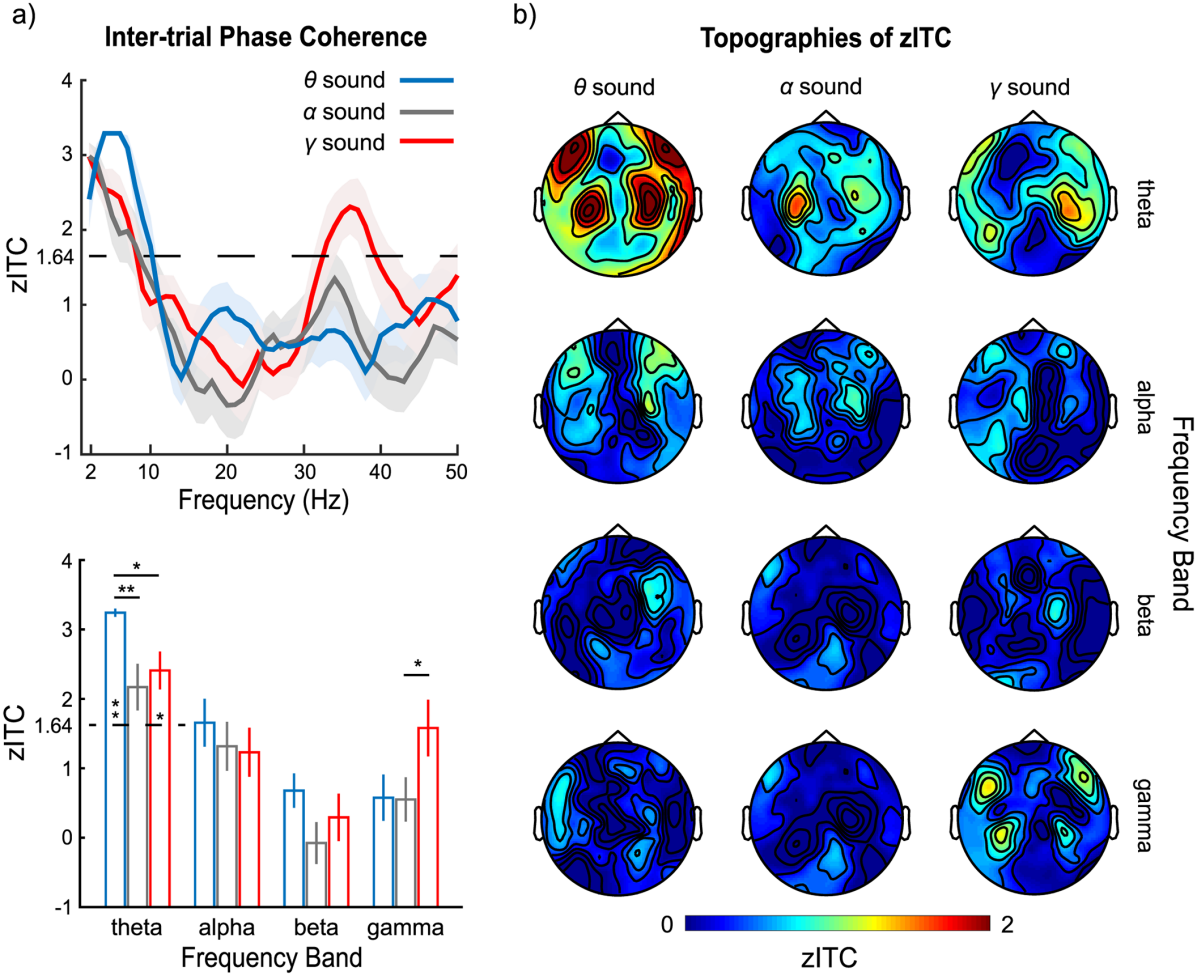
*Z*-scores of intertrial phase coherence (zITC) to 3 clean stimuli and their response topographies. (a) Top panel, spectra of zITC for *θ*, *α*, and *γ* sounds. The dashed line (*z*-score of 1.64) is equivalent to an alpha level of 0.05 (1-tailed, corrected). The shaded areas represent ±1 standard error of the mean. The bottom panel shows zITC for *θ*, *α*, and *γ* sounds in 4 frequency bands, theta (4–7 Hz), alpha (8–12 Hz), beta (13–30 Hz), and gamma (31–45 Hz). The color scheme of blue, gray, and red in both panels represents zITC of *θ*, *α*, and *γ* sounds, respectively. The error bars represent ±1 standard error of the mean. (b) Topographies of zITC for each sound at each frequency band. Auditory response patterns (compared to, for example, classic evoked M100 responses) are observed clearly in the theta band for *θ* sounds as well as for the *α* and *γ* sounds. In the gamma band, the topographies show the auditory response pattern only for the *γ* sound. No clear pattern is observed in other frequency bands. Data are deposited in the Dryad repository: http://dx.doi.org/10.5061/dryad.f357r [121].

The zITC values for the clean stimuli across all frequencies are plotted in Fig 2a and are shown as topographies separated by each band in Fig 2b. ITC, as a general measure of entrainment, shows that the neural theta band (4–7 Hz) is strongly coherent to *θ* sounds and that the neural gamma band (31–45 Hz) is strongly coherent to *γ* sounds. Unexpectedly, the alpha band (8–12 Hz) reveals no selective response to the temporally corresponding stimuli. However, the *α* and *γ* sounds also evoked robust phase coherence at a frequency range below 8 Hz, in which zITC of the group means for *α* and *γ* sounds are above 1.64—the critical *z*-score equivalent to an alpha level of 0.05 (1-tailed, Bonferroni corrected).

Crucially, no robust phase coherence was found in the alpha and beta bands. This nonuniform phase-response pattern across temporal regimes does not support the simplest hypothesis of comparable neural tracking for all stimulus modulation rates. Consistent results were obtained in the spatial analysis. The topographies of zITC, as shown in Fig 2b, reveal clear auditory response patterns both for *θ* sounds in the theta band and for *γ* sounds in the gamma band. Such auditory response patterns, though weak, were also observed in the theta band for *α* and *γ* sounds.

To measure the effects of different stimuli on zITC, a Stimulus-type × Hemisphere × Frequency band 3-way rmANOVA was performed. This revealed main effects of Stimulus-type (*F*(2,28) = 3.88, *p* = 0.033, η_p_^2^ = 0.217) and Frequency band (*F*(3,42) = 30.50, *p* < 0.001, η_p_^2^ = 0.685) as well as an interaction between Stimulus-type and Frequency band (*F*(6,84) = 3.67, *p* = 0.003, η_p_^2^ = 0.208). The main effect of Hemisphere was not significant (*F*(1,14) = 0.22, *p* = 0.648, η_p_^2^ = 0.015).

Planned post hoc comparisons using paired *t* tests with Bonferroni correction on the main effect of Stimulus-type show that zITC of *θ* sounds is larger than zITC of *α* sounds (*t*(14) = 3.36, *p* = 0.014, *d* = 0.87). Post hoc analysis of the main effect of Frequency band with Bonferroni correction shows that zITC at the theta band is larger than the alpha band (*t*(14) = 7.39, *p* < 0.001, *d* = 1.91), beta band (*t*(14) = 9.47, *p* < 0.001, *d* = 2.45), and gamma band (*t*(14) = 7.43, *p* < 0.001, *d* = 1.92) and that zITC at the alpha band is larger than the beta band (*t*(14) = 3.52, *p* = 0.021, *d* = 0.91).

Post hoc analyses on Stimulus-type × Frequency band interactions using adjusted false discovery rate (FDR) correction [67,68] show that, in the theta band, zITC of *θ* sounds is larger than both zITC of *α* sounds (*t*(14) = 4.00, *p* = 0.007, *d* = 1.14) and *γ* sounds (*t*(14) = 3.86, *p* = 0.010, *d* = 1.00); in the gamma band, zITC of *γ* sounds is larger than zITC of *α* sounds (*t*(14) = 2.93, *p* = 0.044, *d* = 0.76). Before adjusted FDR correction, zITC of *γ* sounds is significantly larger than *θ* sounds at the gamma band (*t*(14) = 2.20, *p* = 0.022, *d* = 0.57). There are no significant differences of zITC across different sounds found in the alpha and beta bands after adjusted FDR correction.

To investigate whether *θ*, *α*, and *γ* sounds evoke robust phase coherence in the frequency bands other than their corresponding frequency bands, a 1-sample *t* test of zITC in comparison with a baseline of 1.64 was conducted in each frequency band for each sound. zITC above 1.64 means that the phase coherence observed is above the 95th percentile of ITC distribution over trials of randomized onset time. After adjusted FDR correction, we found robust phase coherence in the theta band for *θ* sounds (*t*(14) = 38.06, *p* < 0.001, *d* = 9.82) and for *γ* sounds (*t*(14) = 3.01, *p* = 0.028, *d* = 0.78) but not for *α* sounds (*t*(14) = 1.66, *p* = 0.240, *d* = 0.43). There are no significant results in alpha, beta, and gamma bands. zITC of *γ* sounds in the gamma band is not significant (*t*(14) = −0.15, *p* = 1.00, *d* = −0.03), likely because robust phase coherence peaks within a narrow frequency range centered around 37 Hz, as shown in the spectrum of zITC in Fig 1a. Averaging zITC of *γ* sound from 30 to 45 Hz decreases the mean of zITC in the gamma band.

To summarize this first set of analyses, we showed that cortical oscillations in the theta and gamma bands, but not in the alpha band, robustly entrained to sounds with modulation rates in the corresponding frequency ranges. The further analysis of zITC indicates that theta band oscillations reliably respond to *γ* sounds whose modulation rate is in the gamma band range. zITC for *α* sound in the theta band also shows phase coherence, although it is not significantly above threshold.

This preferential 2-scale (theta and gamma) response pattern of the cortical auditory system shown by phase coherence results aligns with the findings in neurophysiology related to speech perception and production [69] (although nonspeech stimuli were used here). These consistent results across different stimulus types motivate the hypothesis that 2 discrete timescales, 150–300 ms (theta band) and approximately 30 ms (gamma band), play an important role in general auditory processing. The robust phase coherence observed in the theta band for all 3 sounds further suggests that the phase coherence reflects more than passive entrainment to modulated sounds. Although the alpha band has been found in many auditory tasks to correlate with auditory perception, we did not find robust and distinct entrainment in the alpha band here. It is possible that the stimulus-evoked activity in the alpha band does not manifest in phase but in power, or that alpha does not show preference to any sounds and can be equally entrained by all 3 sounds. Next, we therefore analyzed power responses to 3 sounds. In the classification analysis shown subsequently, we investigated whether phase patterns in each frequency band provide critical information for processing *θ*, *α*, and *γ* sounds.

### Analysis of evoked power responses shows robust but differential entrainment

Having first quantified phase coherence patterns, we next tested whether neural response power reveals patterns that support the observed segregation across bands, as the power response may be differentially modulated by different sounds in specific frequency bands, which could reflect power coding for temporal information. We analyzed evoked power and induced power separately, because evoked power is conceived as a stimulus-locked response while induced power is often argued to be generated by nonstimulus-locked processes [70,71].

**Fig 3:**
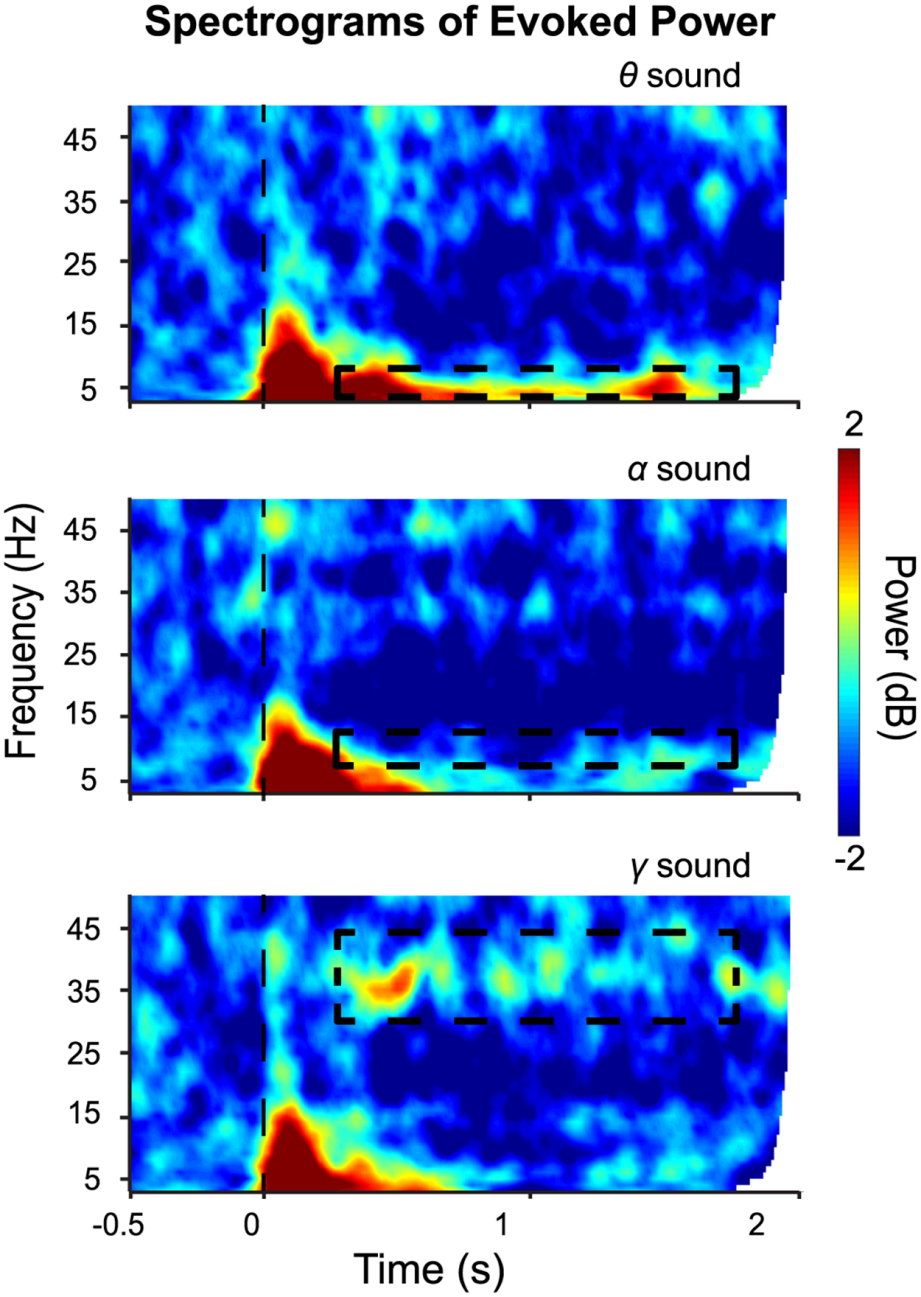
Time-frequency analyses of evoked power. Evoked power responses to *θ*, *α*, and *γ* sounds, respectively. The vertical dashed lines indicate the onset of the auditory stimuli. The dashed boxes in the panels mark the frequency bands of evoked power responses corresponding to the stimulus modulation rates. *θ* sounds evoke power increase in the theta band and *γ* sounds in the gamma band. Data are deposited in the Dryad repository: http://dx.doi.org/10.5061/dryad.f357r [121].

The spectrograms of evoked power for each clean stimulus are plotted in Fig 3. The *θ* and *γ* sounds show corresponding responses in the MEG signal, but the *α* sound does not: the power evoked by *θ* sounds largely distributes in the theta band; the power evoked by *γ* sounds distributes in the gamma band. In contrast, the power evoked by *α* sounds is not well observed in the alpha or any other frequency band.

A Stimulus-type × Hemisphere × Frequency band 3-way rmANOVA was performed and revealed main effects of Stimulus type (*F*(2,28) = 4.29, *p* = 0.024, η_p_^2^ = 0.234) and Frequency band (*F*(3,42) = 28.84, *p* < 0.001, η_p_^2^ = 0.673) as well as an interaction between Stimulus-type and Frequency band (*F*(6,84) = 2.88, *p* = 0.013, η_p_^2^ = 0.171). The main effect of Hemisphere was not significant (*F*(1,14) = 0.33, *p* = 0.575, η_p_^2^ = 0.023).

Post hoc paired *t* tests with Bonferroni correction on the main effect of Stimulus-type show that the evoked power of *θ* sounds was larger than that of *α* sounds (*t*(14) = 3.58, *p* = 0.009, *d* = 0.92). Post hoc analysis of the main effect of Frequency band showed that the evoked power in the theta band is larger than that in the alpha band (*t*(14) = 3.88, *p* = 0.010, *d* = 1.00), in the beta band (*t*(14) = 8.84, *p* < 0.001, *d* = 2.28), and in the gamma band (*t*(14) = 5.14, *p* < 0.001, *d* = 1.33). Evoked power in the alpha band is larger than the beta band (*t*(14) = 5.23, *p* < 0.001, *d* = 1.35), and evoked power in the gamma band is larger than beta band (*t*(14) = 6.32, *p* < .001, *d* = 1.64).

A post hoc analysis of the Stimulus-type × Frequency band interaction using adjusted FDR correction shows that, in the theta band, the evoked power of *θ* sounds is larger than the evoked power of *α* sounds (*t*(14) = 4.77, *p* < 0.001, *d* = 1.23) and *γ* sounds (*t*(14) = 3.32, *p* = 0.020, *d* = 0.86). In the gamma band, the evoked power of *γ* sounds is larger than the evoked power of *α* sounds (*t*(14) = 3.94, *p* = 0.006, *d* = 1.02). There is no significant difference between evoked power across different sounds found in alpha and beta bands after adjusted FDR correction.

Power responses induced by the different stimuli were explored by a Stimulus-type × Hemisphere × Frequency band 3-way rmANOVA. The main effect of Frequency band was significant (*F*(3,42) = 15.36, *p* < 0.001, η_p_^2^ = 0.523). A post hoc analysis with Bonferroni correction showed that the power at the beta band was less than the theta band (*t*(14) = 6.88, p < 0.001, *d* = 1.78), alpha band (*t*(14) = 5.26, *p* = 0.001, *d* = 1.36), and gamma band (*t*(14) = 5.77, p < 0.001, *d* = 1.49). There is no difference in power between theta, alpha, and gamma bands. The main effect of Hemisphere was marginally significant (*F*(1,14) = 4.51, *p* = 0.052, η_p_^2^ = 0.244), with the power in the right hemisphere larger than in the left hemisphere.

### Neural responses in the theta and gamma bands lock to the temporal structure of *θ* and *γ* sounds, respectively

The selective phase coherence observed in the theta and gamma bands that we show in Fig 2 may be a result of reliable auditory responses to any sounds, but not necessarily caused by the specific temporal structure of the stimuli. We therefore asked next whether phase patterns of cortical oscillations actually correlate with the temporal structure of the stimuli. We first used a measure, cochlear-scaled correlation, inspired by the concept of cochlear-scaled entropy, to extract salient acoustic changes that may reset the phase of cortical oscillations and therefore lead to robust phase coherence across trials [51]. The cochlear-scaled correlation was calculated using a moving temporal window and represents acoustic changes along time (see Materials and methods for details). Next, we computed mutual information between the phase series of cortical oscillations at each frequency and the cochlear-scaled correlations. Mutual information can quantify how much information in the temporal structure of the stimuli can be explained by the phase patterns of cortical oscillations and indicate whether the robust phase coherence across trials observed in the neural frequency bands is evoked by the temporal structure of a specific sound.

**Fig 4:**
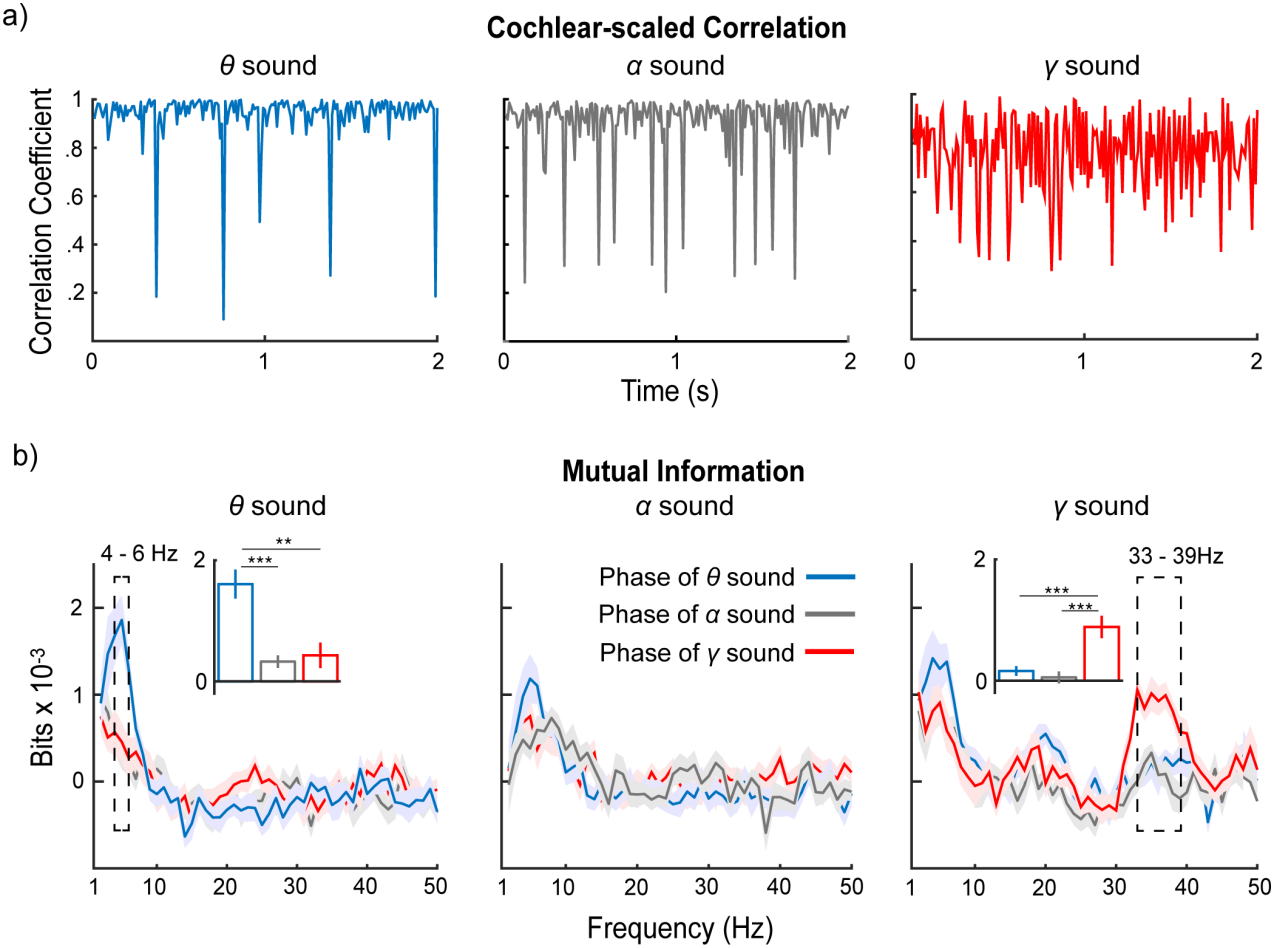
Mutual information between cochlear-scaled correlation and phase of neural oscillations. (a) Cochlear-scaled correlation of *θ*, *α*, and *γ* sounds. (b) Mutual information results. The colors represent mutual information computed using the phase of the neural oscillation to different sounds: blue, *θ* sound; gray, *α* sound; red, *γ* sound. The inset bar graphs show the averaged mutual information within frequency ranges that show significant main effects of the stimulus type. The robust phase coherence observed in the theta band for *θ* sound and in the gamma band for *γ* sound is caused by tracking acoustic structures instead of simply being evoked by general acoustic stimuli. The shaded areas represent ±1 standard error of the mean. Data are deposited in the Dryad repository: http://dx.doi.org/10.5061/dryad.f357r [121].

To compute the cochlear-scaled correlation, we decomposed the stimuli using a Gammatone filterbank with 64 bands, then averaged the amplitude of the envelope in each cochlear band using a moving temporal window of 10 ms, generating 64 total values (1 per band). A Pearson’s correlation was then calculated for these values between each adjacent time point. The cochlear-scaled correlation for each sound is shown in Fig 4a. We computed mutual information between the cochlear-scaled correlation of each sound and the phase series of the neural oscillation to all 3 sounds. For example, we computed mutual information between the cochlear-scaled correlation of the *θ* sounds and the 3 phase series evoked by *θ*, *α*, and *γ* sounds. We used the phase series evoked by *α* and *γ* sounds as controls to examine whether mutual information between the cochlear-scaled correlation of *θ* sounds and the phase series evoked by *θ* sounds is significant. The results of mutual information analysis are shown in Fig 4b.

We ran a 1-way rmANOVA on mutual information with the factor of phase responses evoked by sound type (phase sound type). After adjusted FDR correction across frequencies, we found significant main effects of the phase sound type between 4 and 6 Hz using the cochlear-scaled correlation of *θ* sounds (*p* < 0.05) and significant main effects of the phase sound type between 33 and 39 Hz using the cochlear-scaled correlation of *γ* sounds. Importantly, no significant main effects of the phase sound type were found for mutual information computed using the cochlear-scaled correlation of *α* sounds. In the post hoc comparison with Bonferroni correction, we averaged mutual information within the frequency ranges in which significant main effects were observed and found that, when the cochlear-scaled correlation of *θ* sounds was used, the mutual information computed using phase series of *θ* sound is significantly larger than that using phase series of *α* sound (*t*(14) = 5.29, *p* < 0.001, *d* = 1.37) and that using phase series of *γ* sound (*t*(14) = 3.97, *p* = 0.004, *d* = 1.03). When the cochlear-scaled correlation of *γ* sounds was used, the mutual information computed using phase series of *γ* sound is significantly larger than that using phase series of *θ* sounds (*t*(14) = 5.05, *p* < 0.001, *d* = 1.30) and that using phase series of *α* sounds (*t*(14) = 5.71, *p* < 0.001, *d* = 1.47).

The results of the mutual information analyses demonstrate that phase patterns in the theta and gamma bands track the temporal structure of the stimuli, as quantified by the cochlear-scaled correlation. The robust phase coherence observed in the theta band for *θ* sounds and that in the gamma band for *γ* sounds is indeed caused by tracking specific acoustic structures, rather than simply being evoked by generic acoustic stimuli. The fact that no significant results were found for *α* sounds suggests, again, that the alpha band may play a different role in processing sounds, and especially their temporal structure. In contrast, the theta band and the gamma band may be central to auditory processing and the construction of neural representations underlying perceptual analysis.

We did not find specialized tracking for *γ* sounds in the theta band, although *γ* sounds evoked robust phase coherence in the theta band (Fig 2a). This could be because the theta band, instead of faithfully coding the temporal structure of *γ* sounds, chunks acoustic information and forms a perceptual unit on a timescale of approximately 200 ms (the theta band range). We further explored the contribution of the theta band to coding temporal information of *γ* sounds in the following classification analyses.

### Phase series: Classification analyses and the primacy of the neural theta band

We next performed classification analysis to investigate whether information in each frequency band of cortical oscillations can be used to classify different stimulus types. If a frequency band, for example, the theta band, reflects sufficient information to classify *θ*, *α*, and *γ* sounds, it implies that the theta band plays an important role in processing *θ*, *α*, and *γ* sounds. On the other hand, if a frequency band does not contribute to classifying any sounds, it may indicate that this frequency band is not a key component to processing the sounds.

We first use phase and power responses in a frequency range of 4–45 Hz and a time range of 300–1,900 ms (i.e., after the onset of stimuli) for classification to test whether phase or power provide information related to the temporal structure of each sound. Second, we measure the contribution of each frequency band in classification to determine which frequency band is by hypothesis critical to auditory processing. Finally, we use a new method to classify stimulus type at each time point to investigate temporal progression of the classification performance.

**Fig 5:**
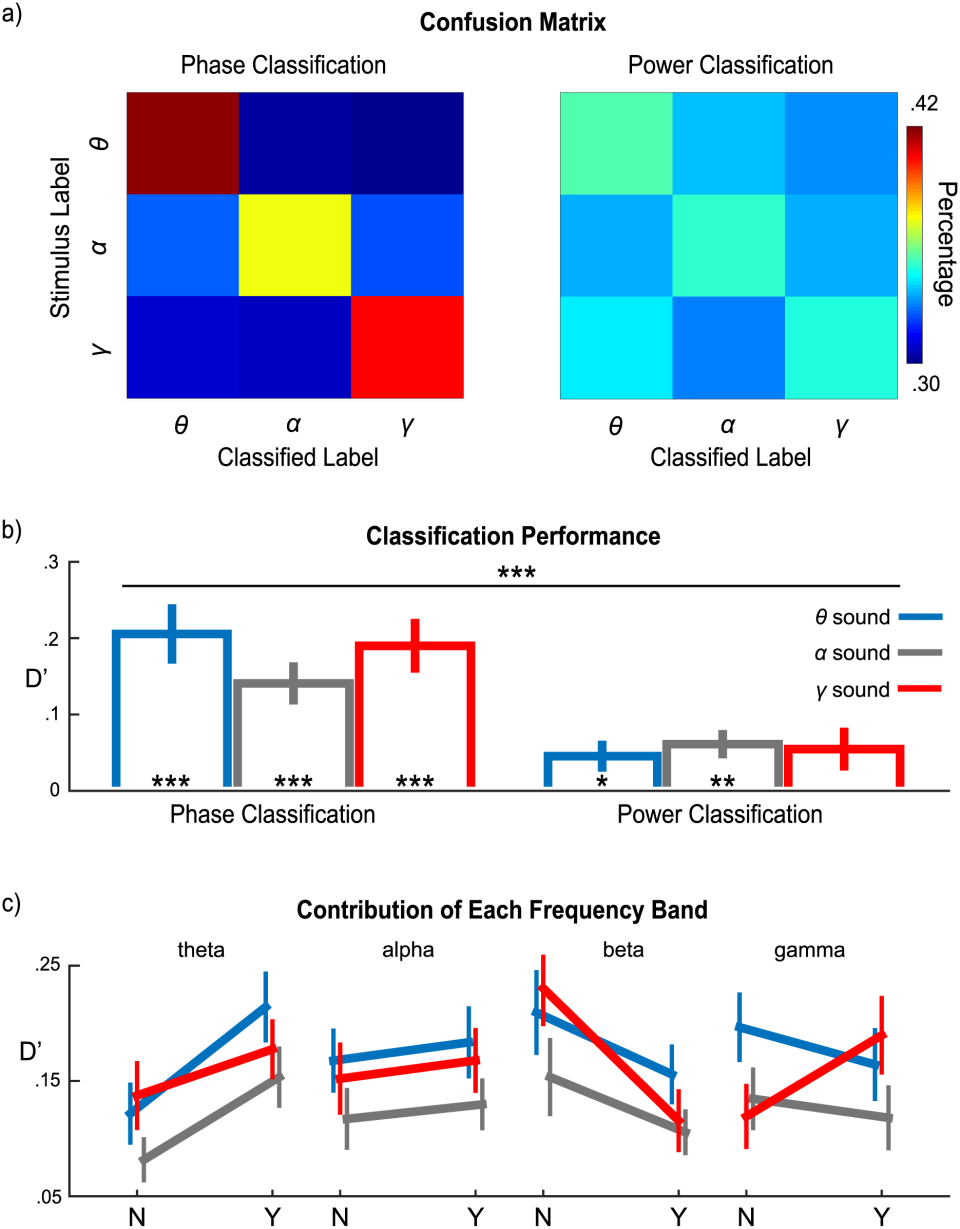
Classification results. (a) Confusion matrices for phase-based and power-based classifications. “Stimulus label” represents the actual stimulus type and “classified label” represents the classified bin. Color bar codes the percentage of trials classified into each bin. (b) Classification performance for each sound using phase and power. Phase-based classification performs significantly better than power-based classification. The blue, gray, and red bars represent *θ*, *α*, and *γ* sounds, respectively. (c) Frequency band contributions to phase-based classification of each sound. “N” indicates classification without the given frequency band; “Y” indicates classification with the given frequency band. Phase in the theta band improves performance for classifying all stimuli and phase in the gamma band contributes to classifying γ sound. The color scheme is as in (b). The error bars represent ±1 standard error of the mean. Data are deposited in the Dryad repository: http://dx.doi.org/10.5061/dryad.f357r [121].

Classification performance was first computed for each stimulus type using the phase and power response profile of all frequency bands (4–45 Hz). Confusion matrices of phase classification and power classification are plotted in Fig 5a. d-prime values computed based on the confusion matrices, indicated by D′ (to differentiate it from *d′* in the behavioral results), are shown in Fig 5b. A Stimulus-type × Classification source (phase classification or power classification) 2-way rmANOVA reveals the main effect of Classification source (*F*(1,14) = 30.44, *p* < 0.001, η_p_^2^ = 0.685), with the D′ of phase-based classification significantly larger than the D′ of power-based classification. To determine whether classification performance is better than chance (D′ = 0), a 1-sample *t* test with Bonferroni correction was applied on each stimulus type and each classification source. For phase classification, the performance of all stimulus types was better than chance (for *θ* sounds, *t*(14) = 6.19, *p* < 0.001, *d* = 1.60; for *α* sounds, *t*(14) = 6.40, *p* < 0.001, *d* = 1.65; for *γ* sounds, *t*(14) = 6.44, *p* < 0.001, *d* = 1.66). For power classification, classification performance of *α* sounds was significant (*t*(14) = 4.73, *p* = 0.002, *d* = 1.22) as well as *θ* sounds (*t*(14) = 3.10, *p* = 0.046, *d* = 0.80), but performance of power classification was only slightly above chance. These results demonstrate that phase patterns of cortical oscillations reliably encode the temporal dynamics of stimuli.

Next, to explore how each frequency band might contribute to phase-based classification of different stimulus types, classification was conducted using different combinations of frequency bands (e.g., theta band plus gamma band, etc.). We then compared the classification performance that was obtained with or without a particular frequency band included in the analysis. For example, to quantify the theta band contribution, we obtained 2 values by either averaging D′ values across frequency band combinations that included the theta frequency band, or without the theta frequency band. The contributions of each frequency band to different stimuli are plotted in Fig 5c. A Stimulus-type × Frequency band × Inclusion (with or without a given frequency band) 3-way rmANOVA shows main effects for Frequency band (*F*(3,42) = 6.28, *p* = 0.001, η_p_^2^ = 0.310) and Inclusion (*F*(1,14) = 16.34, *p* = 0.001, η_p_^2^ = 0.539). The 2-way interactions between Frequency band and Inclusion (*F*(3,42) = 6.28, *p* = 0.001, η_p_^2^ = 0.310) and between Stimulus-type and Frequency band (*F*(6,84) = 5.42, *p* < 0.001, η_p_^2^ = 0.279) were significant as well as the 3-way Stimulus type × Frequency band × Inclusion interaction (*F*(6,84) = 5.42, *p* < 0.001, η_p_^2^ = 0.279). To further examine how each frequency band contributes to classification for each stimulus type, paired *t* tests with adjusted FDR correction were performed on each frequency band and each stimulus type. The theta band contributes to the decoding of all stimulus types (for *θ* sounds, *t*(14) = 8.16, *p* < 0.001, *d* = 2.11; for *α* sounds, *t*(14) = 3.68, *p* = 0.010, *d* = 0.95; for *γ* sounds, *t*(14) = 3.16, *p* = 0.021, *d* = 0.82). The beta band deteriorated decoding of *γ* sounds (*t*(14) = −4.66, *p* = 0.002, *d* = 1.20). Crucially, the alpha band did not contribute significantly to decoding any stimuli. Before adjusted FDR correction, the gamma band shows a contribution to decoding *γ* sounds (*t*(14) = 2.07, *p* = 0.057, *d* = 0.53). After removing 1 subject who showed abnormal decoding performance, we found that the gamma band significantly contributes to decoding *γ* sounds after FDR correction (*t*(13) = 3.35, *p* = 0.017, *d* = 0.90).

**Fig 6:**
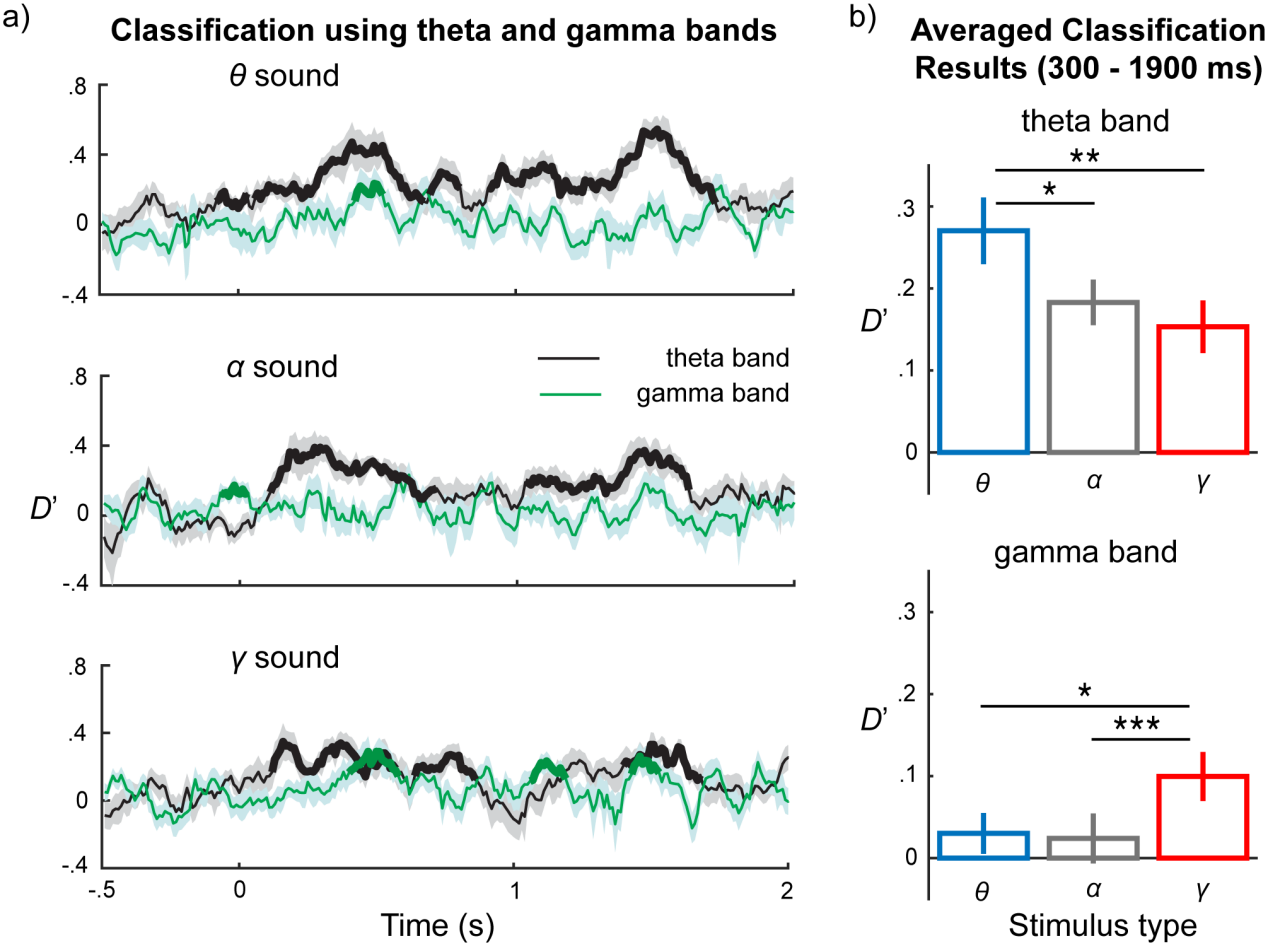
Temporal progression of classification performance in the theta and the gamma bands. (a) Classification performance along each time point. Upper panel: classification results using phase series of the theta and the gamma bands for *θ* sounds, from 500 ms before the onset of stimuli to 2,000 ms after the onset of stimuli; middle panel shows results for *α* sounds; lower panel shows results for *γ* sounds. Classification results using the theta band in black and the gamma band in green. Lines represent the mean classification results averaged across subjects. The **bolded parts** represent clusters significantly larger than baseline, D′ = 0. Significant clusters are observed in the theta band after the onset of stimuli for all 3 sounds. In the gamma band, the significant clusters are found mainly for *γ* sounds. (b) Classification results averaged from 300 to 1,900 ms after the onset of stimuli for each band. The upper panel represents the classification results using the theta band for *θ*, *α*, and *γ* sound and the low panel the classification results using the gamma band. Classification performance for *θ* sounds using the theta band is significantly larger than that for *α* and *γ* sounds, whereas classification performance for *γ* sound using the gamma band is larger than that for *θ* and *α* sounds. The error bars and shaded areas represent ±1 standard error of the mean. Data are deposited in the Dryad repository: http://dx.doi.org/10.5061/dryad.f357r [121].

Finally, because we found that the theta and gamma bands provided the main contributions to classification, we examined how classification performance in the theta band and gamma band progress temporally by using each time point of a phase series to classify a stimulus type. Classification was conducted in the theta band and the gamma band separately by combining the MEG channels selected from 500 ms before the onset of the stimuli to 2,000 ms after. We used a cluster-based permutation test to quantify significance of classification performance (see Materials and methods for details). The results are shown in Fig 6a. We then averaged classification performance for each sound on each band from 300 to 1,900 ms after the onset of stimuli (Fig 6b) and found that in the theta band, classification performance for *θ* sounds is significantly larger than that for *α* sounds (*t*(14) = 3.06, *p* = 0.027, *d* = 0.79) and *γ* sounds (*t*(14) = 4.04, *p* = 0.006, *d* = 1.04) after Bonferroni correction. In the gamma band, classification performance for *γ* sounds is significantly larger than for *θ* sounds (*t*(14) = 2.74, *p* = 0.048, *d* = 0.71) and *α* sounds (*t*(14) = 4.79, *p* < 0.001, *d* = 1.24) after Bonferroni correction.

The significant classification performance in the theta band after the onset of stimuli for all stimulus types demonstrates that the theta band not only entrains to sounds with corresponding modulation rates but also provides critical information for classifying stimuli of all modulation rates. The gamma band showed significant classification performance for *γ* sounds; by averaging time points from 300 to 1,900 ms, we see significantly higher classification performance in the gamma band for *γ* sounds than for *θ* and *α* sounds. This argues for a higher degree of specificity for gamma tracking.

To summarize the classification results: phase series in the theta band temporally track acoustic dynamics across all modulation rates (used in our study), which suggests that the theta band is not only entrained by modulation rates with corresponding frequency range but also chunks sounds with modulation rates outside of the theta band range into acoustic segments at a timescale corresponding to the theta period range. The gamma band specifically locks to modulation rates with a timescale corresponding to gamma band, approximately 30 ms. The contribution of the alpha band must be seen as functionally separate from the other bands.

If, broadly speaking, the (paired) activity of the theta and gamma bands is associated with the construction of perceptual objects in audition, contrary to the alpha band, it stands to reason that the localization of the theta and gamma neural activity should be associated. The supporting information (see S1 Text) provides additional new data to verify that the neural sources are overlapping by localizing the MEG-recorded activity in source space based on individual participants’ structural MRIs.

### Correlation between behavioral results and neural markers

Connecting back to the behavioral data (Fig 1b), we tested how the degraded behavioral performance induced by noise is correlated with the neural markers (ITC and power response) to indicate which neural marker may account for the behavioral results. The positive correlation between *d′* and ITC was significant in the theta band for 3 stimuli (for *θ* sounds, r = 0.815, *t*(14) = 8.50, *p* < 0.001, *d* = 2.19; for *α* sounds, r = 0.378, *t*(14) = 3.50, *p* = 0.014, *d* = 0.90; for *γ* sounds, r = 0.417, *t*(14) = 3.64, *p* = 0.014, *d* = 0.94). The positive correlation between *d′* and evoked power was significant in the theta band for *θ* sounds (r = 0.622, *t*(14) = 8.20, *p* < 0.001, *d* = 2.12). Analysis of the correlation between *d′* and induced power response showed a negative correlation. A significant negative correlation was found in the alpha band for *α* sounds (r = −0.370, *t*(14) = −3.01, *p* = 0.049, *d* = −0.78) and in the gamma band for *γ* sounds (r = −0.347, *t*(14) = −3.22, *p* = 0.048, *d* = 0.83). Adjusted FDR correction was applied to all tests.

The ITC in the theta band showed significant correlation with behavioral performance on recognizing all 3 sounds. These results echo the classification results and demonstrate that the phase series in the theta band provides critical information for auditory processing.

## Discussion

In this MEG-based neurophysiological experiment, we investigate temporal coding at different scales by exploring the entrainment of auditory cortical oscillations to sounds with different modulation rates. Because healthy listeners (appear to) perceive sounds that contain modulation rates over various temporal scales in a manner that reflects a continuous MTF with a low-pass filter shape [16,72], the most straightforward hypothesis suggests that different auditory stimulus rates are tracked in a comparable manner across modulation rates.

Contrary to this hypothesis, we find that oscillations in the (slower) theta and (faster) gamma bands reliably track acoustic dynamics—but not in the (intermediate) alpha and beta bands. Subsequent analyses showed that the information carried in the neural theta band contributes to the decoding of all modulation rates used in this study, whereas the gamma band mainly contributes to decoding only gamma-modulated sounds. Moreover, intertrial coherence in the theta band correlates with identification performance across all stimuli, underscoring that there is a clear perceptual consequence of the entrained oscillatory activity.

Our results are consistent with previous work showing entrainment of auditory cortical activity to low acoustic modulation rates [31,32,45–47,50,51,53,54,57,73–81]. The present results lend support from a new perspective to those studies using modulated sounds with amplitude modulation rates less than 10 Hz that have found strong entrainment in the delta and theta bands. Importantly, the failure to observe entrainment in the alpha and beta bands in the present study also aligns with previous electrophysiological data of monkey primary auditory cortex: cortical oscillations could entrain to modulation rates in the delta-theta bands but not at 12 Hz [73]. Preliminary MEG data have also revealed such a response pattern [66].

The neural gamma band, in addition to the delta and theta bands, also reliably codes temporal information. The finding that the neural gamma band is entrained by sounds with a corresponding temporal modulation rate—and that this alignment contributes to the classification of *γ* sounds—indicates that the auditory system can track acoustic dynamics over short timescales, approximately 30 ms. This observation is consistent with studies using amplitude modulation created by binaural beats; in that work, strong entrainment both in the theta and gamma bands is found—but, again, not in the alpha or beta bands [82]. Recordings in the primary auditory cortex of monkeys also show a phase-locked response using amplitude modulation at 30 Hz [83,84]. The data we show also confirm the contribution of gamma band entrainment to speech separation found in the multiple talker environments [57]. These studies, complemented by the data shown here, support the emerging view that the auditory system extracts precise temporal information mainly on 2 discrete, segregated timescales.

The classification results we report show that the phase information of theta band oscillations contributes to decoding sounds with modulation rates not only at the theta band timescale but also at the alpha and gamma scales. One possible explanation is that theta band oscillations (i) track acoustic dynamics at that specific temporal scale and (ii) at the same time actively chunk (at a scale of the mean theta band period) sounds with faster modulation rates, so that acoustic properties over larger scales can be further extracted [85]. The gamma band exclusively contributes to the classification of sounds with corresponding temporal dynamics. This may indicate specific processing at a fine-grained scale for acoustic temporal details. Overall, theta band oscillations may be necessary, although not sufficient, for processing sounds with temporal variations across different scales, and gamma band oscillations may be needed for fine-grained processing.

The classification results also reveal data patterns that have not been shown in previous findings on the MTF. As the MTF shows decreased neural responses with increasing modulation rate, it is plausible to conjecture that temporal coding for acoustic dynamics would also show such a pattern. However, as observed in the classification analysis, the temporal coding capability does not correspond to the magnitude of the MTF—large ITC values for the alpha band do not indicate high temporal coding capability. Some previous findings, though, do show a tendency for a rebound of neural activity in the gamma band [40,41,44], although the magnitude of gamma band activity is small compared with lower frequency bands [45]. The most relevant finding is from Wang et al. [45], in which a robust response to amplitude-modulated sounds of 31.5 Hz was indicated by the percentage of subjects that showed robust auditory steady-state response (aSSR). These results were suggestive, although the study did not test temporal coding of different frequency bands and concluded “the MTF of the low-frequency aSSR generally has a low-pass pattern and only weakly depends on the carrier bandwidth.” Therefore, although we built our current study on assumptions about the MTF, the previous findings could not resolve the question of auditory temporal coding on different timescales or demonstrate that the power of alpha band is preserved (and “reserved”) not for temporal coding but for other auditory cognitive process.

The absence or marked reduction of tracking acoustic dynamics in the alpha band suggests that neural activity reflected in the alpha band may play a different role in audition. In the auditory system, the neural computations reflected in the alpha band signal may be more explicitly involved in auditory attention, working memory, listening effort, or functional inhibition [34,36–39,86], and the alpha band may as such be more related to aspects of auditory perception that differ from constructing the elementary perceptual representations. The alpha band is associated with suppressing activity of cortical areas that are irrelevant for ongoing sensory processing according to tasks [87], which suggests (for the current context) that alpha band oscillations co-occur with and segregate 2 temporal coding regimes (theta and gamma bands) and modulate auditory processing as a top-down process [88]. The alpha band is involved in processing in other sensory systems and has been well established in visual and somatosensory perceptual analysis [89–92]. It will be relevant to further investigate in the auditory domain how the alpha band interacts with theta and gamma domains to comprehend auditory analysis more fully.

The finding that, like alpha band activity, the beta band also does not track acoustic dynamics may reflect that neural oscillations on that scale are reflective of different operations as well. Beta band oscillations have been argued to play a role in predictive coding [93,94]; the task of the present study does not require active prediction. The random phase in the beta band adds more noise to the classification process, so removing the phase information of the beta band actually results in better classification performance.

Our finding of entrainment, and specifically concurrent parallel processing at different scales of the theta and gamma bands, converges with the 2 perceptual time constants often found in behavioral studies [13]. Experiments on temporal integration frequently report a time constant in the (few) hundreds of milliseconds [14,21–26], while studies examining the high temporal resolution of the auditory system show a time constant less than 30 ms [16–18]. The behavioral results we show also point to higher perceptual sensitivity for theta and gamma sounds compared with alpha sounds. However, the behavioral method used in the present study cannot circumvent a concern with a selection bias to different sounds, because participants may identify *θ* sounds and *γ* sounds more easily simply because the modulation rates of these 2 sounds are located at the perceptual extremes in this experimental design. We acknowledge that this behavioral task is suboptimal—and primarily employed to ensure attention during neurophysiological recording—so we scrupulously refrain from overinterpreting these data, beyond pointing out that the pattern is consistent with our hypothesis. Notwithstanding this potential concern, results from a recent psychophysical study dovetail with the view that the auditory system works concurrently on a short timescale (about 30 ms) to extract fine-grained acoustic temporal detail while processing more global acoustic patterns on a longer timescale (>200 ms) [95].

Importantly, our results, based on nonspeech stimuli, suggest that such dual-scale entrainment is not speech specific but may rather reflect an intrinsic auditory processing property. The auditory cortex tunes to both theta frequency and gamma frequency acoustic dynamics [65]. The alpha band reflects different operations. This segregated, dual tuning of the auditory system at different scales may facilitate the extraction of information of different types in speech, such as featural, segmental, or phonemic information versus syllabic scale information [1]. We suggest that the measured oscillatory patterns at different timescales encode acoustic information over multiple scales, which leads to a temporal multiplexing of sensory information [59,96].

Mounting evidence shows that human perceptual systems employ a discrete process in which continuous signal information is broken up into segments [29,30,97–99]. As natural sounds contain information at multiple scales, the auditory system may chunk continuous sounds using temporal windows of different sizes to sample information at different timescales, instead of processing acoustic information on a unitary scale. This multiplexing strategy solves the requirement in auditory processing that both fine resolution and integration over time are needed for perceiving sounds with regularities both at large and small scales. One model proposes that, although a very high resolution is represented in subcortical areas, in the auditory cortex, there are 2 main temporal windows used for processing acoustic information: one centered around 200 ms and the other around 30 ms [6,100]. On this view, acoustic information is analyzed and integrated using 2 temporal windows at these scales so that perceptual information at such “global” and “local” scales, whether in speech or nonspeech, can be abstracted concurrently to form a unitary percept that forms the basis for perceptual decision-making, lexical access, memory encoding, and other cognitive operations building on elementary perceptual representations. This design, however, builds in a hole in processing, a segregation of function between low and high processing rates—perhaps optimized for sensory sampling—by an intermediate rate, perhaps optimized for allocating attentional and memory resources and functionally inhibiting task- or stimulus-irrelevant actions. Whereas we typically address segregation of function in the spatial domain, i.e., different regions are specialized for different operations, here, we provide a compelling example of cortical segregation of function in the time domain.

## Materials and methods

### Ethics statement

The study was approved by the New York University Institutional Review Board (IRB# 10–7277) and conducted in conformity with the 45 Code of Federal Regulations (CFR) part 46 and the principles of the Belmont Report.

### Participants

Sixteen right-handed volunteers (9 females; mean age: 24.8; standard deviation: 3.2) participated in this experiment. All participants provided informed written consent and received monetary compensation for their participation. Handedness was determined using the Edinburgh Handedness Inventory [101]. All participants had normal hearing and no neurological deficits. We excluded the data from 1 participant because of noise issues during neurophysiological recording. Therefore, the analysis included the data from 15 participants (8 females; mean age: 25.2; standard deviation: 3.0).

### Stimuli and experimental procedure

We created 3 stimulus types following the methods used in Boemio et al. (2005) and Luo and Poeppel (2012). Each stimulus was 2 s long and generated by concatenating narrow-band frequency-modulated segments. The mean starting frequency of each segment was randomly drawn from 2 frequencies, 1,000 Hz and 1,500 Hz. If the mean starting frequency is 1,000 Hz, the frequency-modulated segment could sweep up to 1,500 Hz. If the mean starting frequency is 1,500 Hz, the frequency-modulated segment could sweep down to 1,000 Hz. The bandwidth of segments was 100 Hz (within a critical band at the center frequencies used). We generated each segment by adding up 100 frequency-modulated sinusoids with randomized amplitude and phase. To create a segment that sweeps down, the starting frequency of 100 sinusoid is randomly distributed between 1,450 Hz and 1,550 Hz and the end frequency is distributed between 950 Hz and 1,050 Hz. To create a segment that sweeps up, the starting frequency of 100 sinusoid is randomly distributed between 950 Hz and 1,050 Hz and the end frequency is distributed between 1,450 Hz and 1,550 Hz.

The duration of the segments for each of the 3 stimulus types was drawn from a Gaussian distribution with means of 190 ms, 100 ms, and 27 ms, with standard deviations of 30 ms, 15 ms, and 3 ms, respectively. The distribution of the segment durations of the stimuli aligned with the range of periods typical of theta (4–7 Hz), alpha/low beta (8–15 Hz), and low gamma (30–45 Hz) band neural oscillations. We refer to the stimulus type with mean segment duration of 190 ms as a theta (*θ*) sound, the stimulus type with mean segment duration of 100 ms as an alpha (*α*) sound, and the stimulus type with mean segment duration of 27 ms as a gamma (*γ*) sound. The cochleograms of the 3 stimuli were created using a Gammatone filterbank with 64 banks to decompose the stimuli from 50 to 22,050 Hz [102,103] and are shown in Fig 1a, with the corresponding prior distributions of segment duration for each stimulus type.

We generated white noise segments of 4 s using the random number generator, the function “randn,” in Matlab R2014a (The MathWorks, Natick, MA). Then, we embedded the 3 types of clean stimuli into white noise to create noise-masked stimuli at 5 levels of SNR: −9, −13, −17, −21, and −25 dB. The onset of white noise preceded the onset of the embedded clean stimulus for a random interval uniformly distributed from 1 s to 1.5 s. Thirty stimuli for each SNR level and each stimulus type were created using individually generated noise. As only 1 sample of each stimulus type was generated, the *θ*, *α*, and *γ* sounds were the same across all conditions. Therefore, there were 18 total conditions that included 3 clean stimuli and 15 (3 types × 5 SNR levels) noise-masked stimuli. In total, 540 trials (18 conditions × 30 trials per condition) were presented. The order of all stimuli was pseudorandomized for each participant. After each stimulus was presented, participants were required to push 1 of 3 buttons to indicate the type of stimulus. Between 1 and 2 s after participants responded, the next stimulus was presented, so that all stimuli were presented at random onset points. Participants were required to keep their eyes open and to focus on a white fixation cross in the center of a black screen.

All stimuli were normalized to about 65 dB SPL and delivered through plastic air tubes connected to foam ear pieces (E-A-R Tone Gold 3A Insert earphones, Aearo Technologies Auditory Systems).

### MEG recording and channel selection

**Fig 7:**
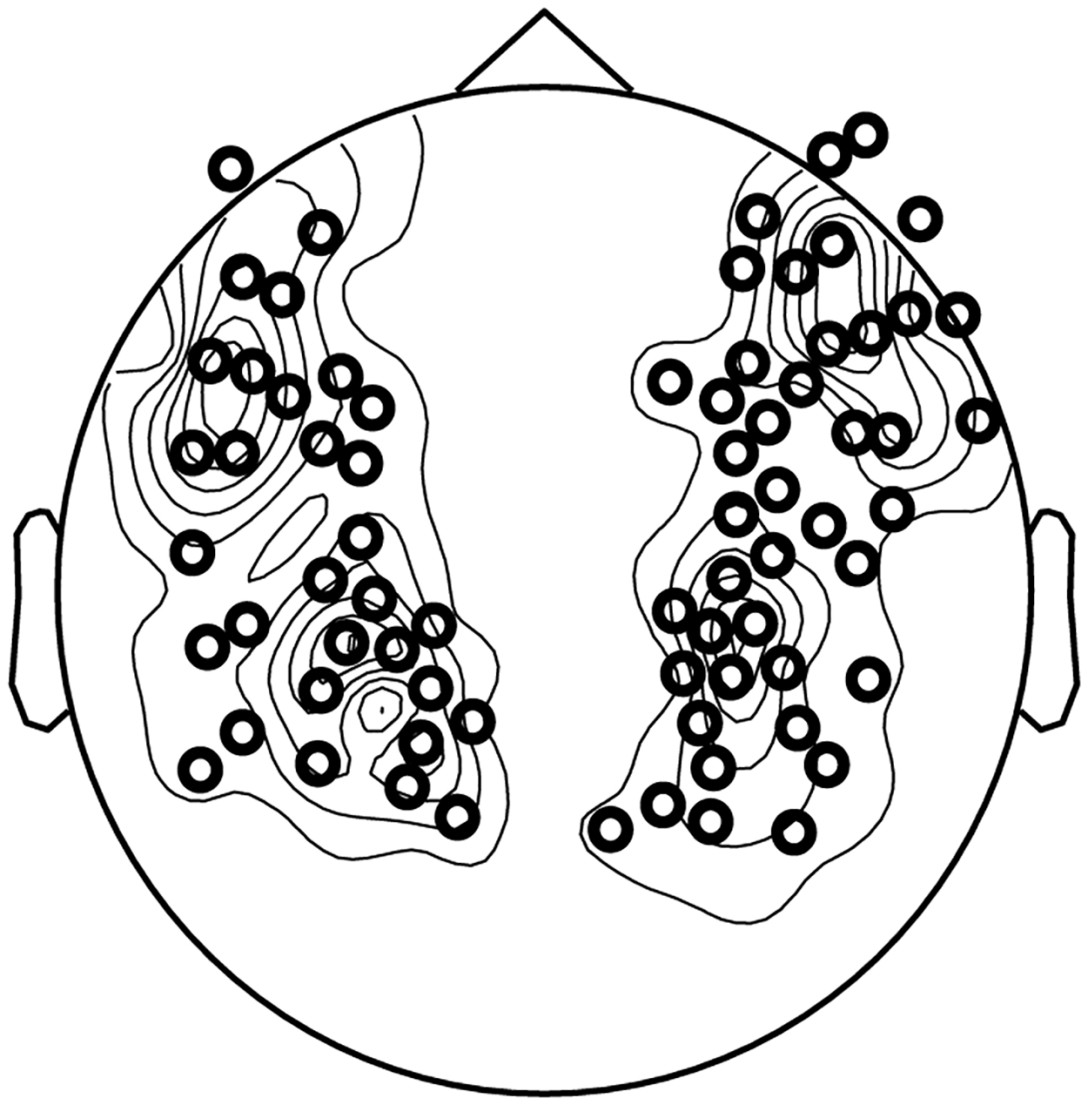
Selected channels pooled across subjects. The plot shows a layout of channels that are selected based on the peak of M100 response across 15 subjects. Twenty channels are selected for each subject (10 in each hemisphere). The channels selected for analysis are indicated by black circles. The contours indicate the extent of overlap across subjects. Data are deposited in the Dryad repository: http://dx.doi.org/10.5061/dryad.f357r [121].

MEG signals were measured with participants in a supine position and in a magnetically shielded room using a 157-channel whole-head axial gradiometer system (KIT, Kanazawa Institute of Technology, Japan). A sampling rate of 1,000 Hz was used with an online 1–200 Hz analog band-pass filter and a notch filter centered around 60 Hz. After the main experiment, participants were presented with 1-kHz tone beeps of 50 ms duration as a localizer to determine their M100 evoked responses, which is a canonical auditory response [104]. Ten channels in each hemisphere, selected based on the peak of M100 response between 60 ms and 120 ms, were used as auditory channels for each participant individually. A layout of channels that are selected based on the peak of M100 response across 15 subjects is shown in Fig 7.

### Behavioral data analysis

Behavioral data analysis was conducted in MATLAB using the Palamedes toolbox 1.50 [105]. For each SNR level as well as the 3 clean stimuli, a 3-by-3 confusion matrix was created and then was collapsed into three 2-by-2 tables by treating 1 stimulus as “target” and pooling the observations on the other 2 stimuli as “noise.” Correct identification of the target stimulus was counted as a “hit” while misidentification of the other 2 stimuli as the target stimulus was counted as “false alarm;” d-prime values were computed based on hit rates and false alarm rates of each table. A half artificial incorrect trial was added to the table with all correct trials [106].

### MEG data preprocessing and analysis

MEG data analysis was conducted in MATLAB using the Fieldtrip toolbox 20140619 [107] and wavelet toolbox. Raw MEG data were noise reduced offline using the time-shifted principle component analysis [108] and sensor noise suppression [109]. Trials were visually inspected, and those with artifacts such as signal jumps and large fluctuations were discarded. An independent component analysis was used to correct for eye blink, eye movement, heartbeat-related and system-related artifacts. Twenty-five trials were included in the analysis for each condition. Each trial was divided into 5-s epochs (1-s prestimulus period and 4-s stimulus period). Baseline was corrected for each trial by subtracting out the mean of the whole trial before further analysis.

To extract time-frequency information, single-trial data for each condition in each MEG channel were transformed using functions of the Morlet wavelets embedded in the Fieldtrip toolbox, with a frequency ranging from 1 to 50 Hz in steps of 1 Hz. To balance spectral and temporal resolution of the time-frequency transformation, from 1 to 20 Hz, the window length increased linearly from 1.5 circles to 7 circles and was kept constant at 7 circles above 20 Hz. Phase and power responses (squared absolute value) were extracted from the wavelet transform output at each time-frequency point.

The ITC, a measure of consistency of phase-locked neural activity entrained by stimuli across trials, was calculated for each time-frequency point (details as in [110]). ITC in different frequency bands reflects phase tracking of cortical oscillations to temporally modulated stimuli. As the baselines of phase-locking may be different across frequency bands, which may be affected by power distributions with a 1/f characteristic, a shuffling method of onset time was used to avoid this confound. Within each condition, the onset time of the stimulus was randomly chosen on each trial and a new dataset for each condition was created. The same analysis of ITC was applied on this new dataset. To create a distribution of shuffled ITCs, this shuffling procedure was repeated 1,000 times. The *z*-score of ITC was computed using the percentile of the original ITC in the distribution.

Induced power was normalized by dividing the mean power value in the baseline range (−0.6 to −0.1 s) and taking logarithms with base 10 and then was converted into values with the unit of decibel by multiplying by 10. The evoked power response was computed by applying the time-frequency transform on averaged temporal responses across all trials. The baseline correction was the same as that used in computing the induced power.

The ITC and power data were averaged from 0.3 s to 1.8 s poststimulus onset to minimize the effects of stimulus-evoked onsets and offsets and within 4 frequency bands: theta (4–7 Hz), alpha (8–12 Hz), beta (13–30 Hz), and gamma (31–45 Hz).

To examine whether phase and power in different frequency bands can explain behavioral performance, correlations between behavioral performance and neural measurements were tested by calculating correlation coefficients between *d′* and either ITC or power across the 5 SNRs, and then a 1-sample *t* test was performed for each frequency band and each stimulus type to determine whether the correlation is significant. As ITC is not normally distributed, the rationalized arcsine transform was applied before calculation of correlation coefficients [111].

All calculations were first conducted in each MEG channel and then averaged across selected auditory channels. Statistical analyses of ITC and power were conducted separately for the 3 clean stimuli and the 15 masked stimuli using rmANOVA. When multiple comparisons were performed, to control familywise error rate and, at the same time, not to cause a high rate of false negatives, the Bonferroni correction was used when there were less than 10 comparisons performed, and an adjusted FDR was used when there were more than 10 comparisons performed [67,68].

### Cochlear-scaled correlation and mutual information

The stimuli in this study were created by concatenating frequency sweep segments of different durations, which may create sharp acoustic edges at the boundary between 2 adjacent segments. These acoustic edges indicate boundaries of frequency sweep segments and represent (one aspect of) the temporal structure of the stimuli, which correlates with modulation rates. By computing mutual information between temporal patterns of these acoustic edges and phase series of MEG signals, we can investigate in which frequency band the neural phase pattern best correlates with the temporal structure of stimuli. The results could tell us whether a frequency band is more or less involved in processing certain stimuli.

To quantify the acoustic edges and extract the temporal structure of the stimuli, inspired by the concept of cochlear-scaled entropy [112], we created an index, cochlear-scaled correlation. We first used a Gammatone filterbank of 64 banks, ranging from 50 Hz to 22,050 Hz, to decompose the sounds and extracted the envelope of each cochlear band. Then, a moving average window of 10 ms was applied on the envelope of each cochlear band to create a vector of length 64 at each time point. We computed the Pearson’s correlation between 2 vectors of adjacent time points and the correlation results were then down-sampled to a sampling rate of 100 Hz, which corresponds to the sampling rate of the phase series of the MEG signals. The cochlear-scaled correlation for each sound is shown in Fig 4a. The visualization shows that cochlear-scaled correlation can confer the temporal structure of stimuli. Within a frequency sweep segment, the cochlear-scaled correlation is high—close to 1—and at the boundaries of frequency sweep segments, a sudden drop of correlation coefficient is evident. The temporal structure of these sudden drops of correlation efficiency correlates with modulation rates, with the *θ* sound having the fewest drops and the *γ* sound having the most drops.

To quantify shared information between the cochlear-scaled correlation and phase series of MEG signals, we used the framework of mutual information [96,113]. Mutual information (MI) was calculated with the Information Breakdown Toolbox in MATLAB [114,115]. We computed the MI between phase series of each frequency (2–50 Hz) extracted from the time-frequency analysis described above and the cochlear-scaled correlation of the *θ*, *α*, and *γ* sounds [50,59,80,116]. For example, when we computed MI between the cochlear-scaled correlation of the *θ* sounds and the 3 phase series evoked, respectively, by *θ*, *α*, and *γ* sounds, we used the phase series evoked by *α* and *γ* sounds as control conditions and examined whether MI between the cochlear-scaled correlation of *θ* sounds and the phase series evoked by *θ* sounds is significant. The mutual information value of each frequency was calculated for each subject and for each channel across trials before averaging.

The cochlear-scaled correlation we compute is simply the values at each time point corresponding to the time point of phase. For each frequency of the MEG response, the phase distribution was composed of 6 equally spaced bins: 0 to pi/3, pi/3 to pi * 2/3, pi * 2/3 to pi, pi to pi * 4/3, pi * 4/3 to pi * 5/3, and pi * 5/3 to pi * 2. By choosing 6 bins for phase information, we ensured that there was enough temporal resolution to capture acoustic dynamics, because (at least) greater than 2 times the temporal resolution than the frequency focused on is needed to quantify information at the frequency. The cochlear-scaled correlation was grouped using 8 bins equally spaced from the minimum value to the maximum value. Eight bins were chosen because we wanted to have enough discrete precision to capture changes in acoustic properties while making sure that each bin has sufficient counts for mutual information analysis, because the greater number of bins would lead to zero counts in certain bins.

The estimation of mutual information is subject to bias caused by finite sampling of the probability distributions because limited data were supplied in the present study (a finite number of trials). Therefore, a quadratic extrapolation embedded in the Information Breakdown Toolbox was applied to correct bias. MI is computed on various subsets of the trials of the dataset of each condition. A quadratic function is then fit to these data points, and the actual mutual information is taken to be the zero-crossing value. This new value reflects the estimated mutual information for an infinite number of trials and greatly reduces the finite sampling bias [117,118].

### Single-trial classification

A single-trial classification analysis of stimulus type was carried out on the clean stimulus condition to examine how the auditory system encodes information at different timescales. The procedure was described in detail in Ng et al. (2013), and similar methods were also used in Luo and Poeppel (2012), Herrmann et al. (2013), and Cogan et al. (2011). For each stimulus type, 1 trial was left out, and then a template was created by averaging across the remaining trials for this type of stimulus (the circular mean is used for phase average). Three templates were created, and the distance between each template and the left-out trial from 1 of the 3 stimulus types was computed. The circular distance was applied for phase classification by taking the circular mean over time and frequency; the l_2_ norm of the linear distance was used for power classification. A trial was given 1 template’s label if the distance between this trial and the template was the smallest among 3 templates.

A confusion matrix of classification was constructed by carrying out classification for each trial of each stimulus type on each auditory channel. Then, classification performance was measured using the same method used in the behavioral data analysis: correctly labeling the target stimulus was counted as a “hit” while labeling the other 2 stimuli as the target stimulus was counted as “false alarm;” *d′* was calculated based on hit rates and false alarm rates and averaged across all auditory channels. Instead of *d′*, D′ was used to differentiate *d′* computed in the classification analysis from *d′* in behavioral results. An index of classification efficiency using phase and power response of difference frequency band was indicated by the mean of D′ over 3 stimulus types, which was compared to the total *d′* of the identification task, which indicates participants’ sensitivity in the behavioral study [106].

### Temporal progression of classification performance in the theta and gamma bands

We carried out classification analysis using only the theta and the gamma bands on each time point to examine how classification performance progresses temporally in 2 frequency bands. We assumed that, on each time point, phase angles across trials can be summarized using a von Mises distribution—the circular analogue of the normal distribution—with its mean approximated by the group mean of phase angles across trials and its kappa value, an index for variance of von Mises distribution, estimated by computing variances of phase angles across trials [119]. For each sound, we calculated the mean and kappa value from 24 out of 25 trials on each time point for 1 sound and left 1 trial out as for classification. The means and kappa values for 3 sounds were estimated and then were used to estimate likelihoods of the left-out trial from each sound belonging to 3 distributions. We computed the likelihoods for each channel and each frequency and summarized the likelihoods across all channels selected and frequencies with each frequency band by adding up log likelihoods of each channel and each frequency. The summarized log likelihood was used to classify the left-out trial. For example, if a left-out trial from a *θ* sound has a high log likelihood in the distribution estimated by 24 trials from *α* sound but lower log likelihoods in the other 2 distributions, we classified this left-out trial from *θ* sound as from an *α* sound.

A confusion matrix of classification was constructed by carrying out classification on each time point in each frequency band for each trial of each stimulus type. Then, classification performance was converted to D′ using the procedure described above in Single-trial classification.

A cluster-based permutation test was conducted on the classification results [120]. For each frequency band, after we assigned the classified labels to the trials from all 3 sounds, we randomly shuffled the classified labels across 75 trials from 3 sounds and created a new dataset of classification results. We then converted the classification results to D′ and conducted 1-tailed 1-sample *t* tests to determine whether D′ on each time point is larger than the baseline line, D′ = 0. We set the threshold of significance as 0.05 and computed cluster-level *t* values of each cluster comprising time points above the threshold. The cluster with the largest cluster-level *t* value was picked for creating a distribution. This procedure was repeated 1,000 times and a distribution over cluster-level *t* values was formed. We set the 95th percentile of the distribution over cluster-level *t* values as the threshold. Then, on the classification results from the original data, we conducted a 1-tailed 1-sample *t* test in each frequency band for each sound and set the threshold as 0.05. The cluster-level *t* values of clusters comprising time points with significant classification performance were computed and the clusters with cluster-level *t* values larger than the threshold determined from the distribution created by permutation were considered as significant clusters. This cluster-based permutation test was conducted in each frequency band for each sound.

Data are deposited in the Dryad repository: http://dx.doi.org/10.5061/dryad.f357r [121].

## Supporting information

S1 Text Magnetoencephalography (MEG) source localization results. (DOCX)

S1 Sound *θ* sound that was used as 1 of 3 stimuli. (WAV)

S2 Sound *α* sound that was used as 1 of 3 stimuli. (WAV)

S3 Sound *γ* sound that was used as 1 of 3 stimuli. (WAV)

## Abbreviations

aSSR: auditory steady-state response
FDR: false discovery rate
ITC: intertrial phase coherence
MEG: magnetoencephalography
MTF: modulation transfer function
rmANOVA: repeated measures ANOVA
SNR: signal-to-noise ratio
zITC: *z*-scores of ITC
