# Supplementary material for: Concurrent temporal channels for auditory processing: Oscillatory neural entrainment reveals segregation of function at different scales
Source: PLoS Biol. 2017 Nov 2;15(11):e2000812. doi: 10.1371/journal.pbio.2000812 (PMC5667736; doi:10.1371/journal.pbio.2000812)
Supplement: S1 Text — (DOCX) [file pbio.2000812.s001.docx]

**Supporting Information**

We provide here a new MEG dataset of 8 subjects (five females, age 21 to 41 years old, right-handed), to show source localization results of the ITC findings for the theta and gamma bands. In the main text we did not provide information on the cortical localizations associated with the robust entrainment in the theta and gamma bands, which potentially leaves a question open: does the robust entrainment within the theta and gamma bands originate from different cortical areas or from the same cortical area (broadly speaking, i.e. within the resolution of MEG, and with the added complication that localizing oscillatory sources comes with further constraints)? Although addressing this question does not impact our conclusion that there exist two discrete time regimes for auditory processing and an intermediate regime reflecting other cortical computations, such localization results do illuminate the neural mechanisms underpinning our findings and strengthen our conclusion.

This new dataset was collected with the stimuli generated using the same procedure and the same parameters as in the main article. ITCs of theta and gamma bands were computed in a similar way and were then projected to source space to show a cortical map of ITC distribution. Fig S1 shows the group-averaged ITCs across all subjects in the theta band evoked by *θ* sounds and in the gamma band evoked by *γ* sounds, separately. From Fig S1 invites the hypothesis that the source localizations of the theta entrainment and the gamma entrainment are very similar, suggesting that theta and gamma band oscillations in the auditory system originate from the same cortical area and concurrently serve as neural substrates to discrete multi-scale auditory processing. This source localization result is consistent with the findings reported in the main text. We append results for individual subjects (Fig S2, S3, S4, and S5).

Fig S1*.* Group average of ITCs across all subjects in the theta band evoked by *θ* sounds and in the gamma band evoked by *γ* sounds. The scale represents ITC values. The source localizations of ITC in the theta and gamma bands are highly similar, which suggests that theta and gamma band oscillations originate from a similar source.

**Stimuli***.* We created five stimulus types with modulation rates distributed in the theta (4-7 Hz), alpha (8-12 Hz), low beta (13 – 20 Hz), beta (20 – 30 Hz), and low gamma (30-45 Hz) bands. For each stimulus type, we generated three sounds with different modulation phases. For example, for the stimulus type with a modulation rate in the theta bands, a *θ* sound, we generated three stimuli with the same modulation rate but with different modulation phase. The procedure used to generate the stimulus types with modulation rates in the theta and gamma bands was the same as in the main text, which is described in details in the Methods. Each stimulus was 2 s in duration.

**MEG recording and experimental procedure***.* The recording procedure was performed as described in the main article. During the MEG recording, participants were required to perform a match-to-sample task: we presented a sample stimulus, which is from one of the five stimulus types, then we presented a pair of ‘match’ stimuli, one of which matched the modulation rate of the sample stimulus. The participants had to choose which one of the stimuli matched the sample. For each stimulus type, we presented each of three sounds 25 times. Only 20 trials for each sound were used to compute the ITC.

**Data analysis**. The source reconstructions were done by estimating the cortically constrained minimum-norm solutions of the MEG data. The forward solution (magnetic field estimates at each MEG sensor) was estimated from a source space of 5124 activity points with a boundary-element model (BEM) method. The inverse solution,was calculated from the forward solution. A high resolution structural T1-weighted MRI scan was acquired for each subject, and the neuromagnetic data were co-registered with the individual brains. Subsequently we morphed each individual brain to the FreeSurfer average brain (CorTechs Labs Inc., Lajolla, CA) and then averaged ITC results across 8 subjects.

We conducted time-frequency analysis and computed ITC using MNE-Python in source space (Gramfort et al., 2014). We computed ITC for each sound using 20 trials and then averaged ITCs from all three sounds for each stimulus type. We selectively analyzed ITC of *θ* sounds in the theta band and of *γ* sounds in the gamma band.

The preprocessed data files are available from the Dryad Digital Repository database: http://datadryad.org/review?doi=doi:10.5061/dryad.f357r

**Reference**

Gramfort, A., Luessi, M., Larson, E., Engemann, D. A., Strohmeier, D., Brodbeck, C., et al. (2014). MNE software for processing MEG and EEG data. NeuroImage, 86 IS -, 446–460. http://doi.org/10.1016/j.neuroimage.2013.10.027

**

Fig S2*.* ITCs of Subjects 1 and 2 in the theta band evoked by *θ* sounds and in the gamma band evoked by *γ* sounds.

Fig S3*.* ITCs of Subject 3 and 4 in the theta band evoked by *θ* sounds and in the gamma band evoked by *γ* sounds.

Fig S4*.* ITCs of Subject 5 and 6 in the theta band evoked by *θ* sounds and in the gamma band evoked by *γ* sounds.

Fig S5*.* ITCs of Subject 7 and 8 in the theta band evoked by *θ* sounds and in the gamma band evoked by *γ* sounds.
